# Raman Submicron Spatial Mapping of Individual Mn-doped ZnO Nanorods

**DOI:** 10.1186/s11671-017-2127-4

**Published:** 2017-05-12

**Authors:** V. Strelchuk, O. Kolomys, S. Rarata, P. Lytvyn, O. Khyzhun, Chan Oeurn Chey, Omer Nur, Magnus Willander

**Affiliations:** 1 V.E. Lashkaryov Institute of Semiconductor Physics, National Academy of Sciences of Ukraine, 45 Nauky pr., 03028 Kyiv, Ukraine; 2 I. Frantsevich Institute for Problems of Material Science, NASU, Krzhizhanovsky str., 3, 03680 Kiev, Ukraine; 3 Department of Science and Technology, Linköping University, 601 74 Norrköping, Sweden; 4 Department of Physics, Kyiv National Taras Shevchenko University, 64 Volodymyrs’ka str., 01601 Kyiv, Ukraine

**Keywords:** ZnO nanorods, Aqueous chemical growth, Optical properties, Raman spectroscopy, Photoluminescence, Magnetic properties, Magnetic force microscopy, Spintronics

## Abstract

ZnO nanorods (NRs) arrays doped with a large concentration of Mn synthesized by aqueous chemical growth and were characterized by SEM, photoluminescence, Raman scattering, magnetic force microscopy (MFM). By comparison of spectra taken on pure and Mn-doped ZnO NRs, a few new Raman impurity-related phonon modes, resulting from the presence of Mn in the investigated samples. We also present a vibrational and magnetic characterization of individual lying nanorods using Raman and MFM imaging. Confocal scanning micro-Raman mapping of the spatial distribution of intensity and frequency of phonon modes in single Mn-doped ZnO NRs nanorods is presented and analyzed for the first time. Mn-related local vibrational modes are also registered in Raman spectra of the single nanorod, confirming the incorporation of Mn into the ZnO host matrix. At higher Mn concentration the structural transformation toward the spinel phase ZnMn_2_O_4_ and Mn3O4 is observed mainly in 2D bottom layers. MFM images of Mn-doped ZnO NR arrays and single nanorod were studied in nanoscale at room temperature and demonstrate magnetic behavior. The circular domain magnetic pattern on top of single nanorod originated to superposition of some separate domains inside rod. This demonstrates that long-range ferromagnetic order is present at room temperature. Aligned Mn-doped ZnO NRs demonstrates that long-range ferromagnetic order and may be applied to future spintronic applications.

**Electronic supplementary material:**

The online version of this article (doi:10.1186/s11671-017-2127-4) contains supplementary material, which is available to authorized users.

## Background

ZnO nanostructures are the wide band-gap semiconductors that exhibit a number of unique properties, which define them as perspective materials for optoelectronic devices such as leds [1], lasers [2], gas sensors [3], UV photedectors [4], Schottky diodes [5], biosensors [6], and spintronics [7]. In the last decade, much attention was focused on ZnO-based diluted magnetic semiconductors (DMS) which retain the spin orientation of charge carriers at the temperatures above room temperature [8]. Theoretical studies [9, 10] predicted the possibility of ferromagnetism ordering at higher temperatures in wurtzite-type GaN and ZnO doped with transition metal ions. Many researchers have studied the long-range magnetic order in DMS and several theoretical models have been proposed, including Zener model [9], bound magnetic-polaron model [11], and models based on spin split impurity bands involving intrinsic defects [12]. It is worth mentioning that a certain amount of defects, such as singly ionized oxygen vacancies (V^+^_O_), can play an important role in the features of the origin of high-T_C_ ferromagnetism in a 3d doped ZnO nanostructures [13–15]. V^+^_O_ centers make the bound magnetic polarons active by exchange interaction, leading to the formation of ferromagnetic domains [11]. However, the origin of the ferromagnetism is still a matter of controversy.

An existence of ferromagnetic behavior at the temperatures up to room temperature (and above) has been observed in many transition-metal doped ZnO films [16], nanowires [17] and crystallites [18]. However, the other authors report that the onset of ferromagnetism is observed upon annealing of samples under low partial pressures of oxygen [19], in zinc vapors [13] or in inert atmosphere [20] and is associated with the formation of clusters of impurity phases [21]. One of the problems for the ferromagnetic ordering in wide band-gap diluted magnetic 3d-doped ZnO semiconductors is low solubility of magnetic impurities. The latter can cause the formation of substantial compositional and structural nonuniformities such as local regions of secondary phase (Mn_3_O_4_ [22] тa ZnMn_2_O_4_ [23]), phase-separation of matrix on the insulating and conducting regions, spinodal fragmentation in the magnetic subsystem etc. [24]. Therefore, for today, the experimental study of direct link between defects and magnetic properties is an important task.

For Mn-doped ZnO magnetic semiconductors, Raman spectroscopy is a fast and powerful technique for studying the microscopic structure, composition, and strain as well as evidence of secondary phase segregation in DMSs [25]. There are a few reports about the study of single nanorod using confocal Raman spectroscopy. Recently, it has been demonstrated that confocal Raman microscopy can be used with the aim to obtain information about phase, growth direction, and radial crystallographic orientation of GaN nanowires [26, 27]. Fan et al. have demonstrated the polarized micro-Raman measurements of single wurtzite CdS nanowires [28]. Yan et al. reported about influence of bending effects on the optical properties of individual ZnO nanowire [29]. In [30], authors reported about observation of quantum confinement effects in a single ZnO nanowire using in situ confocal Raman mapping technique.

In the present paper, we report the study of Mn^2+^ incorporation in ZnO lattice of vertical aligned ZnO nanorods using structural, optical, and magnetic methods. Structural analyses indicated that the nanorod possesses the single-crystalline wurtzite structure. The Raman and PL analysis confirms good crystalline quality of Mn-doped ZnO NRs, which slightly reduces with increases in the Mn concentration. The Raman measurements demonstrated that the substitution of Mn^2+^ in wurtzite ZnO lattice causes an additional mode (AM) at 523 cm^−1^ which is attributed to Mn-related complexes [31]. Mapping of the distribution of Raman intensity and frequency shift of E_2_^H^ phonon mode in the spectra of single Mn-doped ZnO nanorod was also performed to probe the structural quality, strain, and local information about distribution of Mn atoms in ZnO lattice within nanorod. The influence of the effects of Mn doping on the microstructure and magnetic properties of single ZnO nanorod are discussed in detail.

## Methods

Mn-doped ZnO (Mn wt % = 0, 15, and 30) nanorods were prepared by hydrothermal methods with pre-deposited ZnO seed particles on Ag coated sub-micrometer borosilicate glass slides.

The morphology of the samples was investigated using SEM microscopy. Tescan Mira 3 LMU SEM microscope was used.

The XPS core-level spectra of ZnO:Mn films were made in a sublimation ion-pumped chamber of the UHV-Analysis-System (SPECS, Germany) possessing the residual pressure less than 5 × 10^−10^ mbar. The system is equipped with a hemispherical PHOIBOS 150 energy analyzer. The XPS spectra of the studied ZnO:Mn films were excited by Mg Kα source of X-ray irradiation (*E* = 1253.6 eV) and were measured at a constant pass energy of 30 eV. The XPS C 1 s core-level spectrum of binding energy was fixed at 284.6 eV as was suggested for related zinc-bearing compounds [32, 33].

The Raman measurements were acquired in a quasi-backscattering geometry at room temperature using the Horiba Jobin-Yvon T64000 triple spectrometer with integrated micro-Raman setup—Olympus BX-41 microscope equipped with a motorized XYZ stage (minimum step 100 nm) and Peltier-cooled CCD detector. The experiments were carried out using the 488-nm line of an Ar/Kr laser. Low temperature PL spectra were excited using a He-Cd laser with the photon energy hν =3.81 eV.

MFM measurements performed using scanning probe atomic force microscope NanoScope IIIa Dimension 3000™. Two-pass technique was used to eliminate relief influence on MFM data. The MFM tip lift height was 80 nm at second pass. Hard magnetic probes with coercivity of approximately 300 Oe (the NANOSENSORS™ PPP-MFMR probe) were used. MFM tips were magnetized before the measurement along the tip axis in the magnetic field of the permanent magnet. With the aim of better visualization of the samples features revealing the magnetic properties, surfaces were scanned with different polarities of the MFM probe.

## Results and Discussion

### SEM and EDS Analysis

**Fig. 1:**
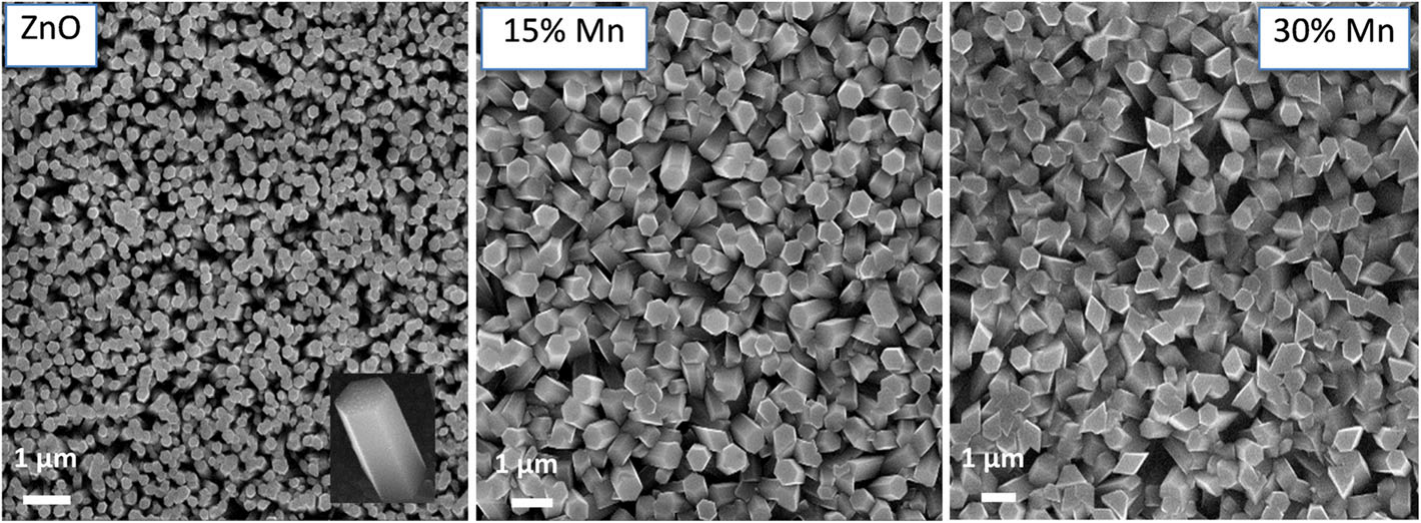
SEM measurements. SEM image of undoped and Mn-doped ZnO nanorods with nominal Mn concentration 0, 15, and 30%

SEM is one of the promising techniques for the topography study of the samples and it gives important information regarding the shape and size of the nanorods. The surface morphology of the undoped and Mn doped ZnO nanorods are shown in Fig. 1. The SEM images clearly show the remarkable increase in the diameter size and change in shape of the nanorods with Mn doping. The SEM image of undoped ZnO NRs (Fig. 1, ZnO) demonstrates the uniform distribution nanorods over the surface. As one can see, undoped ZnO NRs have average diameter close to 250 nm and hexagonal in shape (Fig. 1, inset). When nominal concentration of Mn ions increases to 15 and 30%, the average diameter of the NRs stays almost unchanged within the range of 550 ± 50 nm. Significant deviation in shape of ZnO NRs from the hexagonal facet is observed for 30%Mn sample. NRs with irregular edges and different parts of hexagon side lengths have been shown in Fig. 1. The latter indicates that Mn-doping leads substantially on the morphology of ZnO NRs.

EDS analysis shows that the amount of Mn element in the sample increase in the dependence on nominal Mn concentration in the solution. As a result, Mn incorporation has a strong effect on the optical, structural and morphological properties of ZnO. The results of EDX analysis shown in Table 1 confirm the presence of manganese in the ZnO particles. But wt % is substantially lower compared to nominal value of Mn in ZnO. Also, the certain deviation from stoichiometry corresponding to Zn and O is observed for the undoped NRs.

**Table 1:** The weight % of each element in the undoped and different nominal mole% of Mn-doped ZnO nanorods are obtained from EDS measured for single NRs and array NRs (100 × 100 μm)

Sample	Zn (wt%), single NRs/array NRs	O (wt%), single NRs/array NRs	O(wt%)/Zn(wt%) single NRs/array NRs	Mn (aт.%), single NRs/array NRs
ZnO	52.0/57.3	48.0/42.6	92.3/78.9	-
ZnO:Mn 15%	50.4/11.1	43.4/51.4	86.1/463.1	1.4/8.0
ZnO:Mn 30%	53.2/13.4	43.9/50.7	82.5/378.4	2.4/13.9

EDS measurements, performed on the individual NRs indicate the Mn concentration within the range 0.4…2.4 at.%. The integral EDS measurements from regions sized 100 × 100 μm for NRs arrays and mediated regions, show that for samples with high Mn concentration the substantial increase in the ration O/Zn occurs. The latter can be explained by existence of MnO compounds in the studied samples. Therefore, one can suggest that at high Mn concentrations in NRs or in the 2D bottom layer, except solid solution ZnMnO, the other compounds can forms, in particular manganese oxides.

**Fig. 2:**
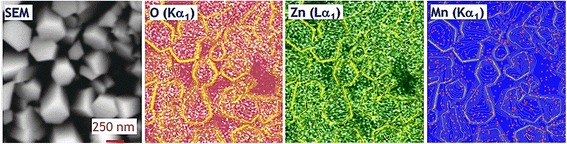
EDS mapping. SEM image of the 30 at.% Mn-doped ZnO NRs and elemental mapping which depicts the distribution of constituents O(Kα_1_), zinc Zn(Lα_1_), and manganese Mn(Kα_1_)

For the determination of the spatial distribution of Mn, EDS mapping was employed. The spatial maps of oxygen and zinc (Fig. 2) correlate with the morphology of the NRs presented on the SEM image, while Mn is almost homogeneously spread element across the sample surface. This means that Mn is incorporated into ZnO matrix of the NRs as well as into underlying 2D bottom layer between the rods. The homogeneous surface distribution of Mn without clustering is important for further arrays of Mn-doped ZnO NRs using in the device applications.

### X-ray photoelectron spectroscopy

**Fig. 3:**
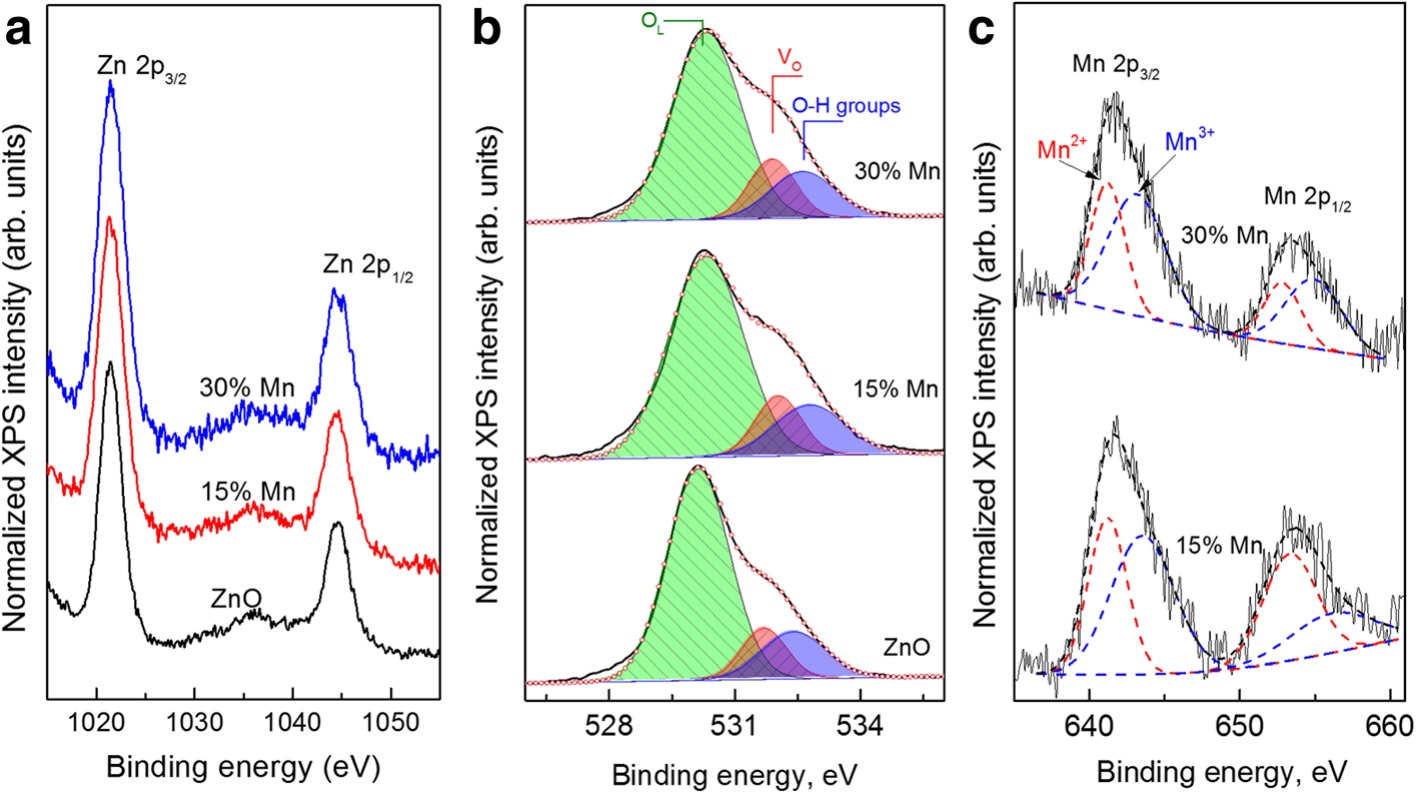
XPS measurements. **a** Zn 2p; **b** O 1 s; and **c** Mn 2p core-level spectra of Mn-doped ZnO nanorods with nominal Mn concentration 0, 15, and 30%

With the aim to analyze the surface valence states of the elements in a nanorods we have used the XPS measurements. Figure 3 shows the XPS spectrum of Mn-doped ZnO nanorods with nominal Mn concentration 0, 15 and 30%. One can see from Fig. 3a, the XPS Zn 2p core-level spectra of the ZnO:Mn NRs are simple spin-orbit doublets formed by the Zn 2p_3/2_ and Zn 2p_1/2_ electrons with binding energies of 1021.4 ± 0.1 and 1044.6 ± 0.1 eV, respectively, due to Zn^+2^ ions [32]. These two peaks have narrow linewidths (~2.7 eV), indicating that the Zn^2+^ ions are dominant in the nanorods. No obvious shift of the Zn 2p peak was observed for the Mn doped ZnO NRs, indicating that the screening effect of electrons (i.e., the charge transfer) from Zn^2+^ ions to Mn^2+^ ions is not significant.

The O 1 s peaks with their deconvolution into three elemental peaks for pure and Mn-doped ZnO NRs are also shown in Fig. 3b. The low-energy peak (O_L_) at 530.3 ± 0.1 eV was attributed to O^2−^ ions surrounded by Zn in the ZnO matrix [34]. The middle peak (V_O_), centered at 531.5 ± 0.1 eV, is associated with oxygen ions in the oxygen-deficient regions within the ZnO matrix and is related to oxygen vacancies [34]. The high-energy (O-H) peak reveals the presence of the fine-structure peculiarities near 532.6 ± 0.1 eV which is usually attributed to chemisorbed or/and dissociated oxygen and OH species on the surface of the ZnO thin film (such as CO_3_, adsorbed H_2_O or O_2_). The presence as well as the changes in the intensity of this component can be related to the variation in the concentration of oxygen vacancies [35].

Figure 3c demonstrates typical Mn 2p XPS spectrum of Mn doped ZnO NRs. Two broad peaks centered at 641.6 and 653.4 eV can be seen and are, respectively, due to Mn 2p_1/2_ and Mn 2p_3/2_, indicating that during the embedding of Mn atoms into the ZnO:Mn NRs lattice, the substitution of the zinc atoms by manganese atoms takes place [36]. The Mn 2p_3/2_ and Mn 2p_1/2_ peaks of Mn doped ZnO NRs could be deconvoluted using two elementary components at 641.2, 643.5, and 653.3, 656.1 eV, respectively. Low energy components with a binding energy of 641.2 and 653.3 eV corresponds to Mn^2+^ ions in the ZnMnO NRs. The high-energy components at 643.5 and 656.1 eV can be attributed to Mn^3+^ and/or Mn^2+^ ions in ZnMn_2_O_4_ (Mn_3_O_4_) of the 2D bottom layer, which are detected by XRD and Raman experiments. In the XPS spectra the metallic Mn ions at 638.7 eV are not detected [37].

With increasing in Mn concentration in the ZnO NRs, the binding energies of the XPS Zn 2p, O 1 s and Mn 2p core-level spectra do not change within the accuracy of the present XPS measurements.

### Photoluminescence

**Fig. 4:**
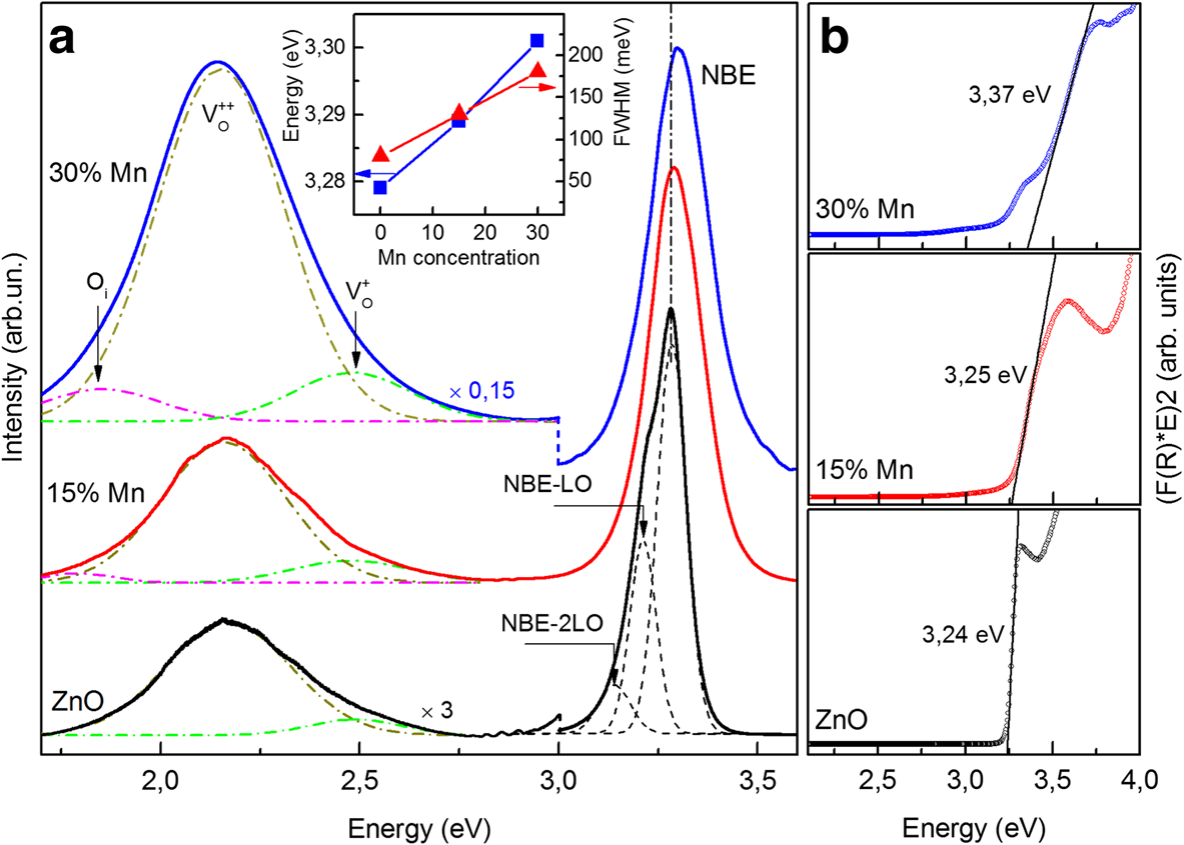
PL investigation. **a** Photoluminescence spectra of Mn-doped ZnO NRs with nominal Mn concentration 0, 15, and 30%. On inset energy position and FWHM of UV emission peak of ZnO NRs on the nominal Mn content. (*T* = 80 K. *E*_exc_ = 3.81 eV); **b** Kubelka-Munk transformed diffuse reflectance spectra of undoped and Mn-doped ZnO NRs

Figure 4a shows the PL spectra of undoped and Mn-doped ZnO NRs. The observed UV peak for undoped ZnO NRs centered near 3.28 eV is attributed to near band edge (NBE) emission of ZnO. In addition, the equidistant maxima at ≈ 3.21 and ≈ 3.14 eV distanced on 72 meV were observed on the short-wavelength wing of the NBE emission in ZnO NRs are attributed to first and second order LO phonon replicas of NBE emission (NBE-LO and NBE-2LO) [38]. With increase Mn concentration in ZnO NRs the NBE emission band intensity decreases and FWHM increases (see Fig. 4a, insertion). Whereas the ratio of UV/VIS intensity decreases from 17 to 0.13. Quenching of NBE emission can be caused by the loss of photo-generated carries through non-radiative transitions to the defect level, which are generated due to Mn incorporation into ZnO crystalline lattice. With increase in Mn concentration, the high-energy shift of NBE emission peak up to ≈ 3.30 eV is observed. This effect is caused by the following facts: (i) the band gap of ZnMnO is higher than that of ZnO [39]*;* (ii) the sp–d spin exchange interaction [40], and (iii) the Burstein-Moss effect [41].

The diffuse reflectance spectra of the pure and Mn-doped ZnO after Kubelka-Munk transformation are shown in Fig. 4b. One can see, that all samples exhibit a strong characteristic absorption peak of ZnO in the ultraviolet region (3.2–3.5 eV). The intersection between the linear fit and the photon energy axis gives the value of E_g_ [42]. The obtained values of energy gap are 3.24, 3.25, and 3.37 eV for 0, 15 and 30% of nominal Mn concentration, respectively. The absorption edge showed the blue shift with increasing in Mn doping concentration indicating enlargement of ZnO:Mn band gap up to 3.37 eV.

However, in the literature also reported about low-energy bandgap shift with increasing concentration of Mn [43–45]. Bylsma et al. [46] explained such reduction in Eg with Mn content by s,p-d exchange interaction between band carriers and d-electrons of Mn^2+^ substituting Zn^2+^ ions. Besides, when the semiconductor is heavily doped, the electrons from the dopant cannot occupy a higher energy level in the conduction band. Thus, these electrons might occupy the energy levels slightly lower than the conduction band due to the band tailing effect, which generates band gap shrinkage. Based on the band tailing effect, the band gap of a heavily doped semiconductor will shrink with the increasing doping levels. XRD measurements of Mn-doped ZnO NRs have shown that with increasing in Mn concentrations the effective expansion of the lattice constants without changing the wurtzite crystal structure occurs; thus, Mn^2+^ substitutes Zn2+ sites (see Additional file 1).

Some previous studies have found out that the green-yellow emission band in PL spectra of our ZnO nanorods should be related to charged oxygen vacancies [47]. As the Mn concentration increases, the relative PL intensity ratio of defect emission to ultraviolet emission in Mn-doped ZnO NRs strongly increases (up to six-times). The defect band emission can be Gaussian fitted with three components, which are centered at 2.46, 2.16, and 1.85 eV, respectively. Generally it is accepted that while the green emission (~2.46) originates from the singly charged oxygen vacancy (V^+^_o_), the yellow one (~2.16) is commonly attributed to the doubly charged oxygen vacancies (V^++^_o_) [47, 48], the red emission (1.85 eV) correspond to oxygen interstitials (O_i_) [47]. The red shift of defect band emission from the 568 nm (2.18 eV) to the 580 nm (2.13 eV) caused by increasing of the contribution of oxygen interstitials (O_i_) in the low energy wing of defect band emission.

Thus, the defect-related bands arise from singly and doubly charged oxygen vacancies and their intensities increase when the population of oxygen vacancies increases after increase Mn concentration in our Mn-doped ZnO NRs. The presence of V^+^_O_ center may be indicate about tend to form bound magnetic polarons, coupling surrounding Mn^2+^ spins within their orbits. With increasing of concentration of V^+^_O_ centers, greater the volume occupied by bound magnetic polarons and larger the probability of Mn ions overlapping leads to a long-range ferromagnetic ordering. When neighboring polarons overlap, the spins of the two-polarons tend to align in the direction of external magnetic field [49]. These effects can be responsible for long-range order ferromagnetism in our Mn doped ZnO nanorods at room temperature.

### Confocal Micro-Raman Spectroscopy

ZnO is a polar crystal and possesses a wurtzite structure with point group C^4^_6V_, both the Zn and O atoms occupying C_3V_ sites. The zone-center optical phonons can be classified according to the following irreducible representations: Γ_opt_ = A_1_ + E_1_ + 2E_2_ + 2B_1_ [50]. The B_1_ modes are silent modes, the *A*_1_ and *E*_1_ modes are polar modes, which are both Raman and infrared active, whereas *E*_2_ modes are non-polar Raman active only. For the backscattering geometry from (0001) crystal plane, only *E*_2_^high^, *E*_2_^low^, and A_1_^LO^ modes are allowed by the symmetry selection rules.

**Fig. 5:**
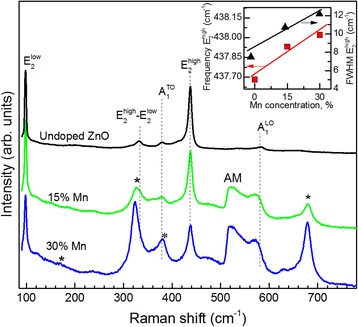
Micro-Raman spectroscopy. Micro-Raman spectra of Mn-doped ZnO nanorods with nominal Mn concentration 0, 15, and 30%. The *asterisks* indicate peaks which correspond to ZnMn_2_O_4_ spinel phase. In the inset, frequency position and full-width of the *E*_2_^high^ mode depending on the nominal Mn content in Mn-doped ZnO nanowires is shown

Figure 5 shows the Raman spectra of undoped and Mn-doped ZnO NRs at the range of 90–750 cm^−1^. For undoped ZnO nanorodes, we have observed common phonon modes centered at 98.3, 332, 379, 437.6 and 584 cm^−1^ corresponding *E*_2_^low^, *E*_2_^high^ - *E*_2_^low^, *A*_1_^TO^, *E*_2_^high^, and quasi-A_1_^LO^ modes, respectively. Both the *E*_2_ and *A*_1_^LO^ modes of wurtzite ZnO are allowed and observed. The *E*_2_^high^ phonon band is downshifted at ~0.6 cm^−1^ compared to the position of *E*_2_^high^ phonon mode in the unstrained ZnO samples (437.0 cm^−1^) [51].

In the Raman spectra of heavily Mn-doped ZnO NRs, the *E*_2_^high^ mode is weak, possibly due to the breaking of translational symmetry caused by intrinsic defects or the dopant. This suggests that the incorporation of Mn into the Zn lattice site in the wurtzite structure of ZnO. As we increase in Mn doping concentration, the *E*_2_^high^ mode slightly high frequency shifted from 437.6 to 438.1 cm^−1^ and the peak broaden asymmetrically from 7.5 to 12.2 cm^−1^ (see Fig. 5, inset).

Another trend apparently connected with increasing Mn concentration, the Raman spectra of 15%Mn-doped ZnO NRs present well-defined peak at 523 cm^−1^, which does not belong to the first- or second-order modes of ZnO. These peak are labeled as AM are obviously related to the manganese impurity. Furthermore, there definitely is a direct correlation between the Mn content and the intensity of this band [52, 53], also observed by us. The appearance of AM for the doped samples can be taken as an indication of Mn incorporation into the ZnO lattice, because these modes were absent in the Raman spectra of the undoped ZnO NRs. Similar AM have already been observed at 525 cm^−1^ for Mn-doped ZnO nanowires and attributed to the presence of Mn ions or Mn-doping-induced defects in ZnO [54]. Recently, Raman measurements of high-quality ZnMnO films grown by OPAMBE revealed two local vibrational modes (LVMs) which is attributed to Mn-(Zinc-vacancy) complexes (at 523 cm^−1^), and the LVM at 712 cm^−1^ is attributed to Mn-(Oxygen-vacancy) complexes [31]. In [55], it is showed that Raman band at 524 cm^−1^ in Mn-doped ZnO is due to the disorder induced activated 2B_1_^low^ silent mode of ZnO. Cong et al. [56] demonstrated that the peak at 524–527 cm^−1^ might be due to the local vibration of the Mn ions at the Zn sites. But the exact the nature of this local vibrations of Mn-related complexes in ZnO is still remains unclear.

Here, we analyze the possibility that Mn-related oxides could be responsible for some Raman peaks present in Mn-doped ZnO NRs with high values of Mn concentration. The increasing of Mn content in the ZnO NRs up to ≥15% leads to the appearance in the Raman spectra the phonon bands at 170, 323, 380, 629, and 679 cm^−1^ (labeled on the Fig. 5 asterisk) which is characteristic of the ZnMn_2_O_4_ spinel phase [57]. The possibility the presence of MnO clusters, for which the LO mode at 552 cm^−1^ [58] is ruled out because the crystal structure of MnO is of the NaCl type.

**Fig. 6:**
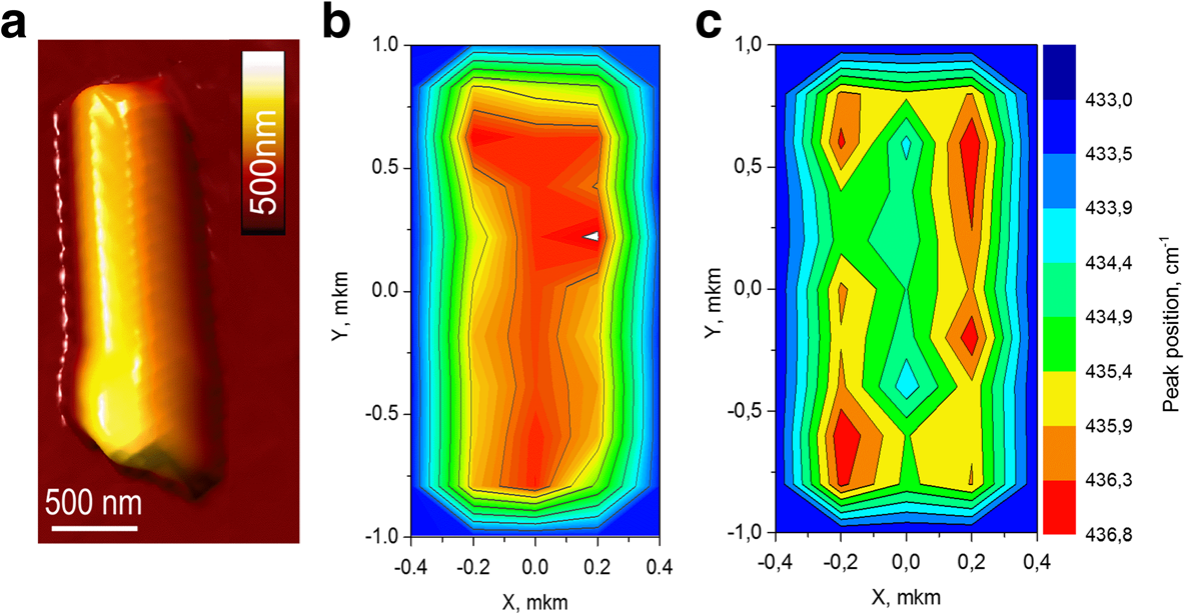
Raman mapping of 15% Mn-doped ZnO. **a** AFM image of 15% Mn-doped ZnO nanorod deposited on an Al-plated mirror. Raman images obtained by extracting (**b**) integrating Raman intensity in region from 415 to 450 cm^−1^ and (**c**) frequency position of *E*_2_^high^ mode of single nanorod

In order to avoid uncertainties where Mn-related oxides formed locally as a result of either a nonuniform distribution or a heavy incorporation of Mn ions in ZnO, confocal Raman mapping spectroscopy was performed on a single Mn doped ZnO nanorod. For carrying out Raman measurements the NRs were extracted from the glass substrate and deposited onto aluminum mirror substrates from the water suspension. In Fig. 6a, it shows an AFM image of a typical ZnO nanorod. Figure 6b, c presents the Raman images of single 15%Mn-doped ZnO nanorod by extracting Raman intensity and frequency position of *E*_*2*_^*high*^ mode. All the Raman spectra were fitted by a Lorentzian profile to determine the Raman peak position and the line width with an accuracy better than 0.2 cm^−1^. The range of contrast scale of Raman images has been fixed within the same frequency range of 433 (blue)-436.8 (red) cm^−1^. As can be seen, even within the nanorod, the frequency of the *E*_2_^high^ mode could differ from one region to the other. This variation is clearly indicated by the frequency distribution in the Raman image (Fig. 6c) mainly due to change in tensile strain and unintentional local impurity doping. With careful observation of the contrast in the Raman image of the nanorod, we noticed that the strain distribution (doping) is not perfectly uniform. The fact that in the center region of nanorod the intensity of the *E*_2_^high^ mode is the strongest (Fig. 6b) and FWHM of *E*_2_^high^ mode is lower correspond to the better crystalline quality.

**Fig. 7:**
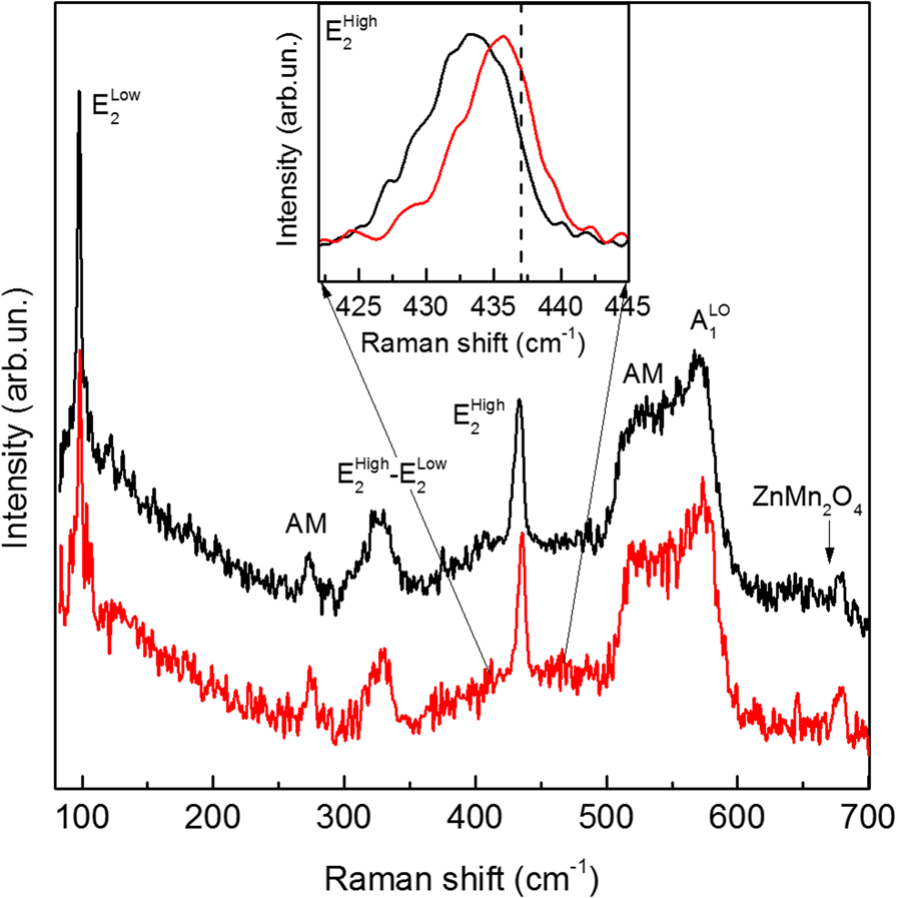
Individual Raman spectra of 15% Mn-doped ZnO nanorod. Confocal micro-Raman spectra of single 15%Mn-doped ZnO nanorod obtained from center (1) and edge of nanorod. The *inset dash line* indicates position of *E*_2_^high^-phonon mode of unstrained ZnO (437 cm^−1^)

As shown in the Figs. 6c and 7, the significant downshift of the *E*_2_^high^ mode over the entire single Mn-doped ZnO NR causes a tensile strain and higher concentration of Mn. Additional Mn-related local vibrational modes are also registered in Raman spectra, confirming the incorporation of Mn into the ZnO host matrix of the single nanorod. More interesting and importantly, the weak Raman signal from spinel ZnMn_2_O_4_ secondary phase is also registered.

**Fig. 8:**
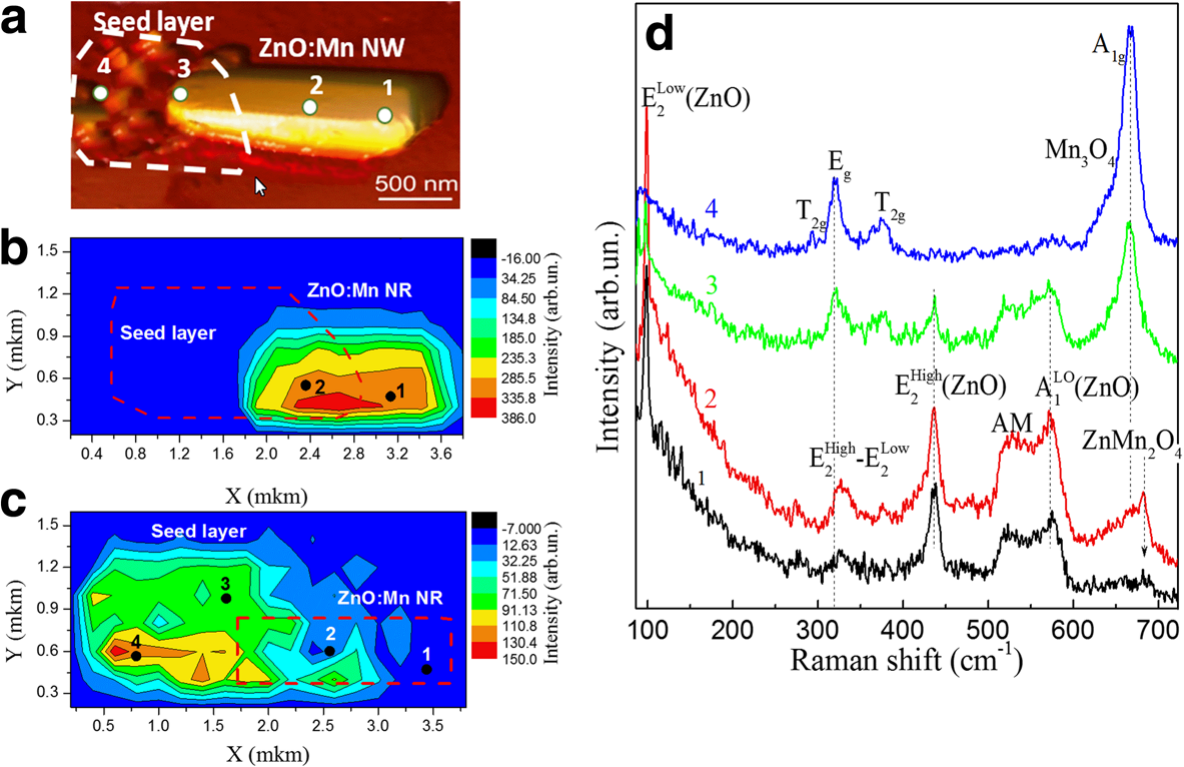
Raman mapping of 30% Mn-doped ZnO. **a** AFM image of individual ZnO:Mn (30%) NR with the part of the bottom seed layer, (**b**) Raman map of *E*_2_^high^ mode intensity along individual nanorod, (**c**) Raman image intensity of A_1g_ mode of the spinel Mn_3_O_4_ (ZnMn_2_O_4_) structure, and (**d**) Raman spectra obtained from different mapped points which shown on Fig. 8b, c. The *dashed lines* in **a** and **b** depict borders of ZnMn_2_O_4_ bottom 2D layers and in (**c**) contour of ZnO nanorods

Somewhat different situation is observed for the single 30%Mn-doped ZnO nanorod with the part of the bottom layer (see AFM image on Fig. 8a). The dashed lines in Fig. 8a, b depict borders of ZnMn_2_O_4_ bottom 2D layers and in Fig. 8c contour of ZnO nanorods. The Raman image of the integrated intensity of the *E*_2_^high^ mode in the region from 415 to 450 cm^−1^ shown in Fig. 8b clearly reveals inhomogeneity distribution intensity over the entire nanorod. The detailed Raman mapping of integrated intensity of the *A*_1g_ mode of the ZnMn_2_O_4_ (Mn_3_O_4_) spinel structure in the region from 640 to 680 cm^−1^ (Fig. 8d) reveals that this secondary structural phase mainly located in the bottom seed layer. On the Fig. 8d shows a series of Raman spectra from various points which shown on Fig. 8b, c along single 30%Mn-doped ZnO nanorod and the part of the bottom seed layer. It can be seen from the Raman spectra of the single nanorod shown on Fig. 8d (curves 1 and 2) that there are all characteristic ZnO phonon modes and Mn-related additional mode at 525 cm^−1^.

Similar to single 15%Mn-doped ZnO nanorod, for the *E*_2_^high^ mode, the frequency is lower and FWHM is smaller in the center region of the nanorod in comparison with the edge region. This is a consequence of better crystalline quality in the center nanorod. When approaching the area of the bottom seed layer, in the Raman spectra of the single 30%Mn-doped ZnO nanorod, the Raman bands of the ZnMn_2_O_4_ spinel structural phase are registered in the region 650–680 cm^−1^. The ZnMn_2_O_4_ has a tetragonal crystal structure in which the Zn^2+^ ions occupy the tetrahedral sites and the Mn^3+^ ions occupy the octahedral sites. The intensity of these modes is significantly increased in the region of the seed layer in the bottom part of the nanorod. Moreover, secondary spinel phase of Mn_3_O_4_ is registered in some local regions of this 2d bottom layer. Thus, in the area seed layer we found Mn_3_O_4_ spinel structure, identified by the Raman spectrum (curves 4), which exhibits five phonon peaks: a (triply degenerate) *T*_2g_ symmetry mode at 290 cm^−1^, a (doubly degenerate) *E*_g_ symmetry mode at 319 cm^−1^, *T*_2g_ symmetry mode at 374 cm^−1^ and a (singly degenerate) *A*_1g_ symmetry “breathing” mode at 665 cm^−1^ [23]. A similar effect was observed in studying mechanism for the structural transformation from ZnO nanobelts into single-crystalline Mn_3_O_4_ nanowires, and creations heteronanostructures consisting of ZnO, ZnMn_2_O_4_, and Mn_3_O_4_ [59].

### AFM and MFM Microscopy

Magnetic force microscopy (MFM) measurements are the direct probing of magnetic properties of individual nanorods. The intrinsic magnetic features of Mn-doped ZnO NR arrays in nanoscale at room temperature were further studied by MFM with a magnetic tip magnetized before the MFM measurement along the tip axis. Images of magnetic field gradients are recorded far enough above the sample surface effectively diminishes the contributions of short-range nonmagnetic Vander-Waals forces acting on the probe so that only the MFM signal is detectable. In MFM mode, the vibrating magnetic probe traces the surface morphology to maintain a constant distance (80 nm) between the surface samples and the tip apex and senses the magnetic tip-surface interaction. Possible influence of electrostatic interactions was effectively reduced by sample discharging prior measurements.

**Fig. 9:**
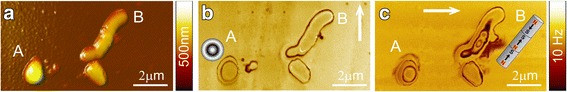
AFM and MFM measurements. AFM (**a**) and MFM (**b**, **c**) images of vertically (**A**) and horizontally (**B**) aligned fragments of ZnO:Mn (15%) nanorode. *Arrows* indicate direction of MFM tip scanning. *Inserts* in **b** and **c** show model sketches of magnetic domains

MFM measurements allow revealing magnetic polarization in a nanorod with high spatial resolution. As clearly proven by the MFM measurement. The top of the nanorod shows a circular domain pattern (fragment A and insert in Fig. 9b) and corresponding magnetic pattern transformation detectible over sidewalls of the nanorod (fragment B in Fig. 9c).

The circular domain pattern on top of nanorods originated to superposition of some separate domains inside. MFM tip induces some remagnetization effects of nanorods depending on scanning direction (parallel or perpendicular to the axis of nanorode) that is anisotropy of single nanorode magnetization. This could be either due to a ferromagnetic coupling or to paramagnetism in the field of the MFM tip. However, since all measurements were performed at room temperature, paramagnetism as the source of the magnetic contrast seems unlikely [60].

**Fig. 10:**
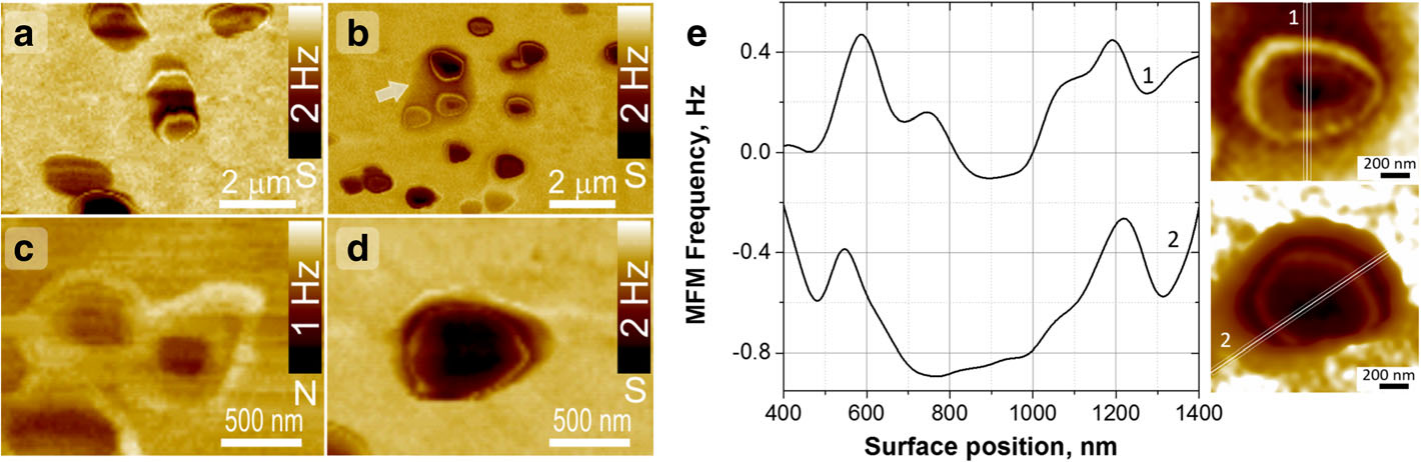
MFM measurements. MFM images of upright standing ZnO:Mn (Mn15%) NRs measured by MFM tip with opposite polarity of apex, north (N) or south (S) (**a**–**d**). *Arrow* in **b** shows 2d bottom layer. **e** Represents diametric profiles of magnetic field gradients (shift of MFM tip oscillation frequency) over top of NRs collected by north (curve 1) and south (curve 2) magnetized MFM tip apex

It is well known that a unique structure feature of the nanostructured materials is their high surface-to-volume ratio. Large fractions of the atoms in nanorods are situated on the surfaces or interfaces. Spin behavior of surface atoms differ considerably from the bulk and in general interface magnetism is likely to differ from bulk behavior [61, 62]. A higher MFM signal is present at the boundaries than in the center of separate nanostructures (Fig. 10). The paramagnetic Mn^2+^ ions possess a positive magnetic susceptibility, meaning they induce a small positive MFM signal when interacting with the tip, while the diamagnetic ZnO gives no signal in MFM [63]. The ring-shaped complex structure with higher MFM magnetic contrast on boundaries of ZnO:Mn NRs is clearly visible. This effect is caused by increasing in Mn-dopant atoms concentration and intrinsic/native defects (oxygen vacancy ets) on the boundaries of NRs that are responsible for the in Mn-doped ZnO. Opposite polarity of MFM tip leads to reversing of MFM contrast from ZnO:Mn-boundaries, i.e., tip-surface interaction changes from repulsion to attraction. Diametric profiles shown in Fig. 10e illustrate positive and negative shift of MFM signal collected by “north” and “south” magnetized tip apex and semi-quantitatively characterize magnetic “strength” of surface and central areas of nanorods. The absolute MFM signal shift is significantly large at the peripheral area of ZnO:Mn-boundaries in comparison with the central area.

Some local areas at the substrate (arrow marked at the Fig. 10b) could be detected with a pronounced magnetic contrast. These flat areas between nanorods looks like clusters of 2d seed layer produces by chemical reactions at nanorods growth. Probably the seed layer can cover sidewalls of nanorods increasing the radial magnetic contrast.

## Conclusions

In this study, we combined SEM, EDS, XPS, photoluminescence, Raman spectroscopy, MFM as reliable methods to investigate the structural, and optical and magnetic properties of the Mn-doped ZnO nanorods synthesized by the hydrothermal method. The effect of the Mn-doping and its concentration on structural, optical, and magnetic properties of Mn-doped ZnO NRs were studied in detail. The incorporation of Mn in ZnO caused significant changes in shape, size, and density of the nanorods. The Raman active vibrational modes in Mn-doped ZnO NRs show peak shifts and broadening as a result the doping-induced relaxation of the symmetry selection rules.

Confocal scanning micro-Raman mapping spectroscopy was performed on a single Mn-doped ZnO nanorod. The Raman spectra of single ZnO:Mn nanorods show the Raman-active vibrational modes of wurtzite phase ZnO and local vibration modes associated with Mn-related complexes at 523 cm^−1^. From the analysis of frequency position of *E*_2_^high^(ZnO) and Mn-related modes, Raman images of spatial distribution strain, crystalline quality of magnetic impurity were analyzed. At higher Mn concentration, strong vibrational modes related to ZnMn_2_O_4_ and Mn_3_O_4_ spinel phase were found in the Raman spectra of Mn-doped ZnO NRs arrays. Both phases coexist mainly in entire array of the 2d bottom seed layer with variable contributions.

Magnetic properties of Mn-doped ZnO NR arrays and single nanorod are studied by magnetic force microscopy imaging, and obtained clear magnetic contrast in nanoscale at room temperature demonstrates a strong evidence of ferromagnetic behavior. The circular domain magnetic pattern on top of single nanorode was observed. This result shown that our method may be applied to future large-scale manufacture of aligned Mn-doped ZnO nanorod arrays with ferromagnetic properties.

## Additional Files

Additional file 1: XRD study. (DOCX 368 kb)

## Abbreviations

AFM: Atomic force microscopy
AM: Additional mode
DMS: Diluted magnetic semiconductors
FWHM: Full width at half maximum
LVM: Local vibrational mode
MFM: Magnetic force microscopy
NRs: Nanorods
PL: Photoluminescence
SEM: Scanning electron microscopy
XPS: X-ray photoelectron spectroscopy
