# Supplementary material for: Raman Submicron Spatial Mapping of Individual Mn-doped ZnO Nanorods
Source: Nanoscale Res Lett. 2017 May 12;12:351. doi: 10.1186/s11671-017-2127-4 (PMC5429288; doi:10.1186/s11671-017-2127-4)
Supplement: Supplementary file 1 — XRD study. (DOCX 368 kb) [file 11671_2017_2127_MOESM1_ESM.docx]

**Raman submicron spatial mapping of individual Mn doped ZnO nanorods**

Strelchuk V. ^1^, Kolomys O.^1^_,_ Rarata S.^1,4^, Lytvyn P.^1^, O. Khyzhun^2^,

Chan Oeurn Chey^3^, Omer Nur^3^, and Magnus Willander^3^

*^1^V.E. Lashkaryov Institute of Semiconductor Physics National Academy of Sciences of Ukraine, 45 Nauky pr., 03028 Kyiv, Ukraine*

*^2^ I. Frantsevich Institute for Problems of Material Science, NASU, Krzhizhanovsky str., 3, 03680, Kiev, Ukraine*

*^3^Department of Science and Technology, Linköping University, 601 74 Norrköping, Sweden*

*^4^Kyiv National Taras Shevchenko University, Department of Physics, 64 Volodymyrs’ka str., 01601Kyiv, Ukraine*

**XRD study**

The XRD patterns of pure and Mn- doped ZnO array nanorods with different nominal Mn concentration are presented in Fig. S1. The XRD spectra revealed (111), (200) and (220) peaks caused by the Ag layer deposited onto the glass substrate. The XRD pattern of the Mn-doped ZnO NRs exhibits three strong diffraction peaks (100), (002) and (101). Both the pure ZnO and Mn doped ZnO NRs show hexagonal wurtzite structures (JCPDS card no. 01-071-5959) with highly preferential their orientation along the c-axis (002). For Mn doped ZnO NRs the (002) diffraction peak shifted to lower angles from 34.403^0^ (pure ZnO NRs) to 34.384^0^ (30 % Mn); the lattice parameter c increased (Fig. S1b). Such changes of the peak position and lattice parameter are to be expected at replace of Zn ions on Mn ions in the lattice because the ionic radii of Mn^2+^ ions (0.66 Aº) are larger than that of Zn^2+^ ions [*P. Singh , A. Kaushal , D. Kaur, J Alloys and Compounds 471 (2009) 11.*]. For high concentration of Mn doped ZnO NRs additional (112), (200) and (103) XRD peaks are observed which correspond to tetragonal ZnMn_2_O_4_ spinel (JCPDS card no. 24-1133) (Fig.S1c). The intensities of these peaks in the XRD pattern increase with increasing nominal Mn concentration.

Fig.S1. (a) XRD patterns of the Mn-doped ZnO array NRs. (b) Lattice parameters “c” and XRD (002)-peak position versus nominal Mn concentration. c) XRD peaks of the ZnMn_2_O_4_ secondary phases for 30%Mn ZnO NRs.
